# Retraction: Systemic Lupus Erythematosus: An Overview of the Disease Pathology and Its Management

**DOI:** 10.7759/cureus.r13

**Published:** 2019-04-16

**Authors:** Asad Ali, Zohaib Sayyed, Muhammad Atif Ameer, Abdul Wahab Arif, FNU Kiran, Ayesha Iftikhar, Waleed Iftikhar, Malik Qistas Ahmad, Muhammad Bilal Malik, Vijay Kumar, Arjan Dass, Shahzad Ahmed Sami, FNU Sapna, Neha Waqas

**Affiliations:** 1 Internal Medicine, CMH Lahore Medical College and Institute of Dentistry, Lahore, PAK; 2 Pediatrics, Shaikh Khalifa Bin Zayed Al-Nahyan Medical and Dental College, Lahore, PAK; 3 Department of Internal Medicine, Lahore Medical and Dental College, Lahore, PAK; 4 Medicine, Shaikh Zayed Hospital, Lahore, PAK; 5 Internal Medicine, Basic Health Unit Larhi, Karachi, USA; 6 Internal Medicine, Nishtar University, Multan, Multan, PAK; 7 Hematology/Oncology, University of Arizona Cancer Center, Tucson, USA; 8 Internal Medicine, Shifa College of Medicine, Lahore, PAK; 9 Internal Medicine, Ghulam Muhammad Mahar Medical College, Sukkur, PAK; 10 Internal Medicine, Burhani Hospital, Karachi, PAK; 11 Surgery, Shaikh Khalifa Bin Zayed Al Nahyan Medical & Dental College, Broken Bow, PAK

This article has been retracted at the request of the Editor-in-Chief as it contains significant plagiarism from Yeo S, Dias S, Isenberg D. Advances in systemic lupus erythematosus. *Medicine*. 2018;46(2):84-92. 10.1016/j.mpmed.2017.11.010

A key condition of article submission is that authors must explicitly declare that their work is original and has not appeared in a publication elsewhere. Re-use of any data must be appropriately cited. This duplication was not detected by Cureus plagiarism check software as much of the material had been rewritten just enough to avoid detection. While the authors have maintained that this was the result of working with a contract editor, they are ultimately responsible for the draft submitted.

As such this article represents a severe abuse of the scientific publishing system. The Cureus Journal of Medical Science takes a very strong view on this matter and we apologize that this was not detected during the submission process. The relevant author institutions and departments have been notified.

